# Feasibility of ultra-low-field MRI in an ion beam treatment room for online MR-guided particle therapy

**DOI:** 10.1016/j.phro.2026.101092

**Published:** 2026-09-26

**Authors:** Julia Pfitzer, Hermann Scharfetter, Panagiotis Antypas, Dietmar Georg, Hermann Fuchs

**Affiliations:** a Department of Radiation Oncology, Medical University of Vienna, Spitalgasse 23, Vienna, 1090, Austria; b Institute of Biomedical Imaging, Graz University of Technology, Stremayrgasse 16/III, Graz, 8010, Austria

**Keywords:** Ultra-low-field MRI, Ion beam therapy, Experimental evaluation, MR-guided ion beam therapy

## Abstract

**Background and purpose:**

Online MR-guided particle therapy may improve beam delivery precision and treatment efficacy. Ultra-low-field (ULF) MRI may enable image guidance with minimal modification of existing facilities. The feasibility of operating a ULF magnetic resonance (MR) scanner for online MR-guided particle therapy in an unmodified ion-beam therapy environment was investigated.

**Material and methods:**

A portable 49 mT ULF-MRI system (OSI^2^ ONE MR scanner) was placed at the isocenter of a research room at an ion-beam therapy facility without radio-frequency shielding. Image quality was assessed using a grid phantom under static and dynamic irradiation conditions for protons and carbon ions. Electromagnetic interference (EMI) was characterized and mitigated via grounding and local shielding. Beam deflections were quantified using a scintillation detector and compared to numerical simulations. The impact of ongoing proton and carbon ion irradiation on the imaging process was assessed. Dosimetric measurements were performed using a Semiflex ionization chamber in a PMMA phantom with and without simultaneous MR imaging.

**Results:**

Stable image quality was achieved across proton and carbon ion irradiation with no relevant irradiation-induced artifacts. Initially substantial EMI was reduced to near-baseline levels by internal shielding. Proton beam deflections ranged from 0.9–1.7 mm at isocenter, consistent with simulations. Dosimetric deviations were below 0.05% for 2D fields and 0.02% for spread-out Bragg peaks (SOBP).

**Conclusion:**

ULF-MRI operation in an ion-beam therapy environment without a dedicated shielded room is feasible, without compromising imaging, beam delivery, or dosimetry. This approach supports future MR-guided particle therapy research with reduced infrastructural requirements.

## Highlights

• For the first time, ultra-low-field MRI was investigated in an ion beam therapy room.
• Image quality was assessed for interference from room, beam, and scanning magnets.
• MR imaging is feasible in an unmodified ion beam therapy treatment room.
• No image degradation or artifacts arose from ion beam delivery or scanning magnets.
• Ion beam delivery and dosimetric measurements are feasible during MR imaging.

## Graphical abstract

**Figure:**
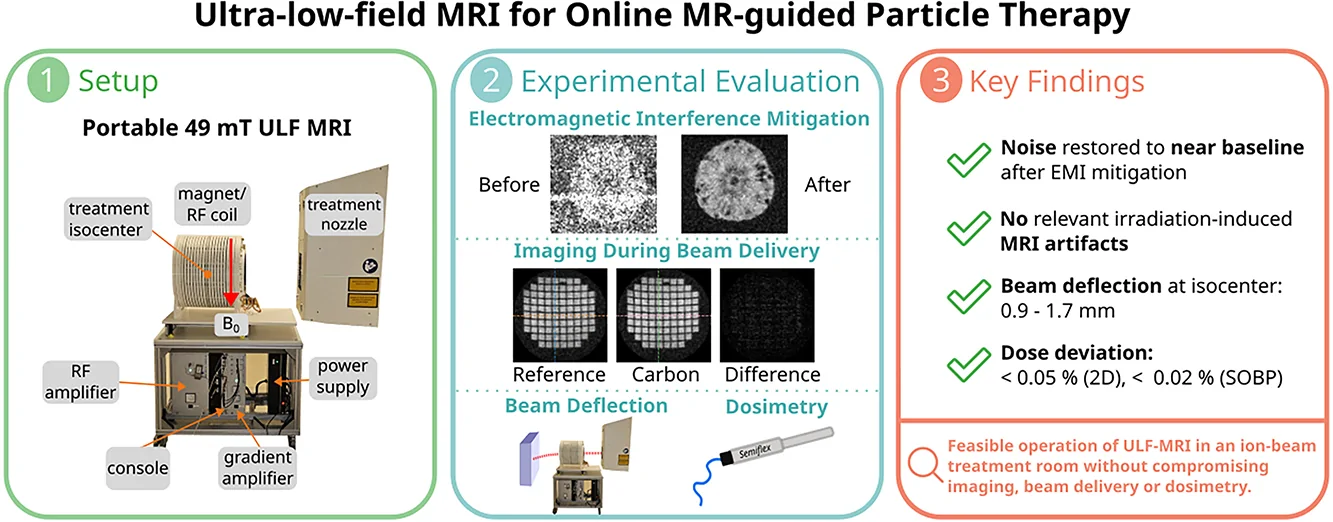

## 1 Introduction

In MR-guided photon radiotherapy based on hybrid MR-linac systems, soft-tissue visualization has enabled margin reduction and a decrease in total irradiated volume, highlighting the potential of MRI-based guidance [1].

For ion-beam therapy, due to the higher conformity and the sensitivity to changes in anatomy compared to photon therapy, the combination of MR and ion beam therapy beam line could be even more beneficial [2] and is the focus of intense research. First prototypes have already been developed employing 0.25 and 0.32 T open-MR scanners in proton or ion-beam therapy treatment rooms [2], [3], [4]. The combination remains highly technically challenging, as the strong magnetic fields required by conventional MR systems interact with the primary treatment beam requiring adaptations to treatment planning, dose delivery, and dosimetric approaches [5], [6], [7], [8], [9], [10], [11].

In addition, MR imaging performance itself is affected by Electromagnetic Interference (EMI) as well as stray magnetic fields originating from beam delivery components, requiring extensive shielding and mitigation strategies to avoid image degradation or artifacts [4], [10], [12], [13].

Ultra-low-field MRI (ULF-MRI), typically operating at magnetic field strengths below 0.2 T, is associated with reduction in Lorentz-force-induced beam perturbations, while simultaneously reducing the complexity of the primary magnet itself, removing the need of expensive superconducting coils, associated quench pipes, as well as considerably lowering the weight compared to high-field MRI systems.

However, reducing the field strength decreases the signal-to-noise ratio (SNR), which may reduce spatial resolution and require longer acquisition times [14]. Nevertheless, the combination of ULF-MRI and ion-beam therapy is a promising alternative for next-generation image-guided particle therapy, enabling MR imaging with minimal impact on existing facilities. Initial studies have begun to address system design and coil requirements [15].

In this work, we experimentally evaluated the feasibility of integrating a ULF-MRI system in an unmodified treatment room of an ion-beam therapy center, for the first time. The potential impacts of surrounding EMI and magnetic fields due to realistic beam delivery on image quality and dosimetry were systematically evaluated.

## 2 Material and methods

### 2.1 Irradiation room

The research room at the MedAustron ion-therapy center (Wr. Neustadt, Austria) is equipped with a horizontal beam line for scanned ion-beam delivery. It features a medical nozzle, which is capable of delivering a $20\times 20\;{\mathrm{cm}}^{2}$ field with protons and carbon ions in the clinical energy range of 62–253 MeV and 120–403 MeV/u, respectively. In addition, the room is equipped with a laser positioning system as well as the same couch mounted X-ray based imaging and positioning system as in the clinical treatment rooms.

### 2.2 ULF-MRI scanner

An ultra-low field MR scanner, based on the OSI^2^ ONE MR scanner design [16], [17] (49 mT, Larmor frequency 2.11 MHz at 22 °C) was built at Graz University of Technology and transported to the MedAustron facility. A full system characterization is provided by Schote et al. The scanner used in this study is the same unit included in their cross-site comparison [17]. The system has previously been used for in-vivo neuroimaging as well as phantom MRI measurements [18], [19].

**Fig. 1:**
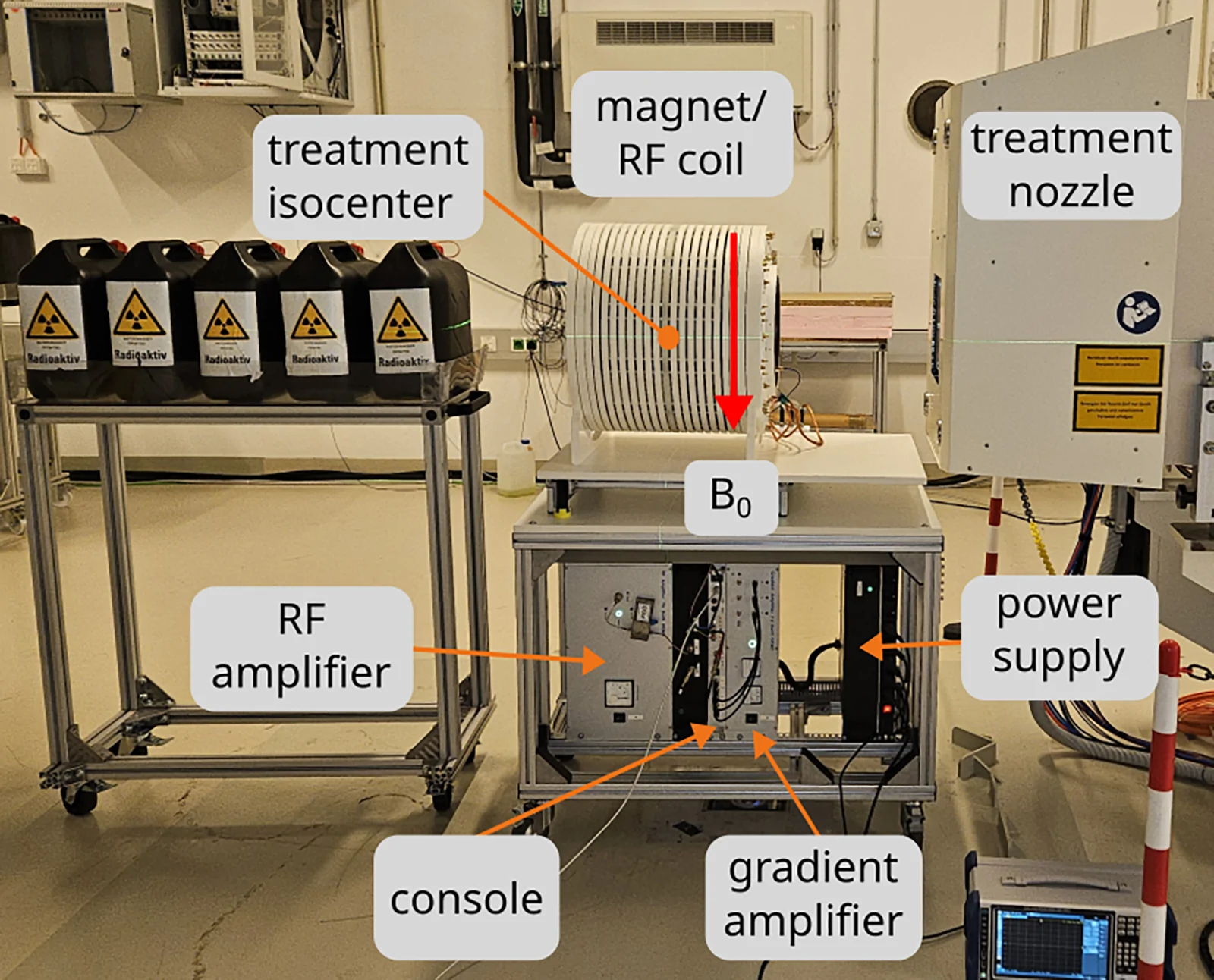
OSI^2^ ONE scanner at the isocenter of the research room, the different scanner components have been annotated. The orientation of the main magnetic field ($B_{0}$) is indicated with a red arrow. The ULF-MRI system includes a wheeled cart on which the magnet is placed. In the cart below the magnet, the electronic system is mounted, making the system fully portable. It operates from a conventional AC power outlet, with a power consumption of 120 W. The system was controlled from the adjacent control room via a network connection.

### 2.3 Setup

The ULF-MRI system was positioned in the irradiation room such that the MR isocenter and the beamline isocenter coincided. The bore of the system was oriented such that the beam had unhindered access into and through the ULF-MRI system (Fig. 1). No external radiofrequency shielding was applied.

To avoid irradiating the ULF-MRI system, especially the RF coil electronics, the maximum field size was limited to $15\times 15\;{\mathrm{cm}}^{2}$ for all treatment beam applications.

### 2.4 Electromagnetic interference (EMI)

EMI mitigation methods are an active area of research in ULF-MRI since at such low field strengths, the body acts as an efficient capacitive coupling pathway for EMI, which can then couple directly to the RF coil. This has a detrimental effect on image quality.

Different passive EMI mitigation strategies include subject grounding [20], conductive blankets placed over patients [21], and shielding structures integrated within RF coils [18]. Active noise cancellation techniques employ additional RF coils positioned around the scanner to detect EMI and subtract these interference signals from the MRI signal [22], [23].

With the presence of patient positioning robots, vacuum pumps, and various other electrical equipment, considerable EMI was expected.

To investigate EMI effects in the patient treatment room, an EMI phantom was constructed. This phantom consisted of a pineapple for imaging, connected to a large aluminum foil surface placed outside the scanner for EMI pickup. The aluminum foil and pineapple were connected with a short cable and a copper needle was inserted into the pineapple. In a previous study a large conductive surface placed outside the magnet which was electrically connected to the imaging phantom was found to be adequate to emulate body-mediated EMI coupling. Experiments confirmed that it closely matched the capacitive coupling mechanism observed in vivo [18].

Environmental EMI was then investigated under three conditions:

• No EMI mitigation (No Grounding, No Shield)
• Phantom grounding (Grounding, No Shield) using a 100 Ω series resistance to mimic an electrode-body interface
• Phantom grounding and electric shielding within the RF coil (Grounding and Shield)

A schematic representation of the experimental setup as well as details on the shielding configuration can be found in the supplementary material.

To quantify environmental EMI levels, spectra were recorded over a 50 kHz bandwidth without any MRI sequence and compared to a 50 Ω baseline where the RF chain was terminated with a 50 Ω resistor instead of the RF coil as described by Guallart-Naval et al. [21]. The comparison to a measured 50 Ω baseline considers only EMI coupled via the RF coil and excludes other pathways, such as conducted interference from internal electronics, which should be mitigated with appropriate filtering and shielding.

Additionally, imaging experiments were performed to observe EMI effects on image quality. SNR was calculated using four noise ROIs placed outside the imaging region and scaled to account for the magnitude images and a central ROI for mean signal estimation. MRI acquisition parameters can be found in the supplementary material. Image reconstruction was performed as described in the imaging section below.

### 2.5 Imaging with and without beam delivery

Calibrations of RF power and Larmor frequency were performed prior to each imaging experiment. The excitation frequency was not adjusted during MRI measurements to compensate for temperature-induced shifts of the Larmor frequency (∼2.5 kHz °C^-1^ [24]). The temperature was not measured during the experiments.

A 3D-printed grid phantom (15 mm spacing, 148 mm x 148 mm outer grid distance) was used for image quality as well as interference evaluations. Imaging was performed with a resolution of $2\times 2\times 5\;{\mathrm{mm}}^{3}$ using a 3D Rapid Acquisition with Relaxation Enhancement (RARE) sequence, covering a $200\times 200\times 120\;{\mathrm{mm}}^{3}$ field of view in approximately 4 min. The full acquisition parameters for the MRI experiments can be found in the supplementary material.

All images were reconstructed using an inverse fast Fourier transform. Images were masked in a circular region of interest and rotated for visualization.

In a first step, imaging of the grid phantom located at the MR center was performed at the isocenter of the irradiation room without ongoing beam delivery.

The difference between the reference and active beam configurations was visualized by computing the absolute difference of the normalized magnitude images. Additionally, a difference image was created from two subsequent reference measurements without beam delivery to show variation of the system between normal acquisitions. The signal intensity was plotted along a central vertical and horizontal line for each comparison. Peak Signal-to-Noise Ratio (PSNR) between reference and beam-delivery was calculated for the different scanning configurations over the masked and normalized image. Geometric accuracy was determined by manually measuring the S-I and L-R dimensions for a central slice. The image quality was assessed using a subjective visual quality assessment as well as calculating the mean SNR over ten slices in the different configurations.

To assess beam delivery effects on image quality, three scenarios were studied: (i) a static central beam, (ii) slow and fast scanning beams in horizontal and vertical directions, and (iii) homogeneous 2D irradiation. The beam delivery was not synchronized with the MR sequence in any of the presented scenarios.

For the static case, a central proton beam at 252.7 MeV with the fastest extraction speed (1 s) was delivered to the grid phantom at isocenter, yielding a flux of $1.6\times 10^{10}$ protons per second.

The potential influence of the scanning magnets was evaluated using 402.8 MeV/u carbon ions in horizontal and vertical lines at about 1 and 100 cm/s, with alternating directions. Discrete spots at 1 cm spacing were irradiated to mimic clinical delivery and introduce dynamic scanning speed variations.

Finally, a homogeneous 2D pattern of $6\times 6\;{\mathrm{cm}}^{2}$ with 2 mm spot spacing was delivered using 148.2 MeV protons, as used for dosimetric evaluations below. All patterns were repeated for the duration of the imaging process.

An overview of the employed irradiation fields can be found in Fig. 4(a).

### 2.6 Beam delivery

The impact of the primary magnetic field on the particle beam trajectory was investigated using a Lynx scintillation detector (IBA Dosimetry, Schwarzenbruck, Germany). The Lynx detector was located behind the ULF-MRI system at a distance of 50 cm downstream of the isocenter, placing the Lynx detector about 28 cm behind the magnet bore.

A five-spot map was created, consisting of a central spot and four corner spots. This spot map was irradiated with monoenergetic proton beams of 62.4, 148.2, and 252.7 MeV, using $1.6\times 10^{8}$ protons per spot.

Measurements were repeated three times. After irradiation, the ULF-MRI system was removed and the measurements repeated. Spot positions as well as size and shape were evaluated and compared with and without ULF-MRI system.

A Runge–Kutta 4th order algorithm ($1\times 10^{-7}\text{mm}$ step size) was employed to numerically solve the effect of the Lorentz force on the primary beam propagation inside the low-strength magnetic field and compared to the experimental trajectories. To simplify calculations, the magnetic field was assumed to be homogeneous over an effective length of 62 cm, incorporating fringe-field effects. The effective field extent was validated against experimental measurements behind the magnet.

In a second step, the same monoenergetic 2D $14\times 14\;{\mathrm{cm}}^{2}$ irradiation field described above was measured with and without the ULF-MRI system. Besides lateral offsets, the field size at 50% and 90% dose level, penumbra width (dose fall-off from 80%–20%), symmetry as well as homogeneity were evaluated.

### 2.7 Dosimetry

An $8\times 8\times 8\;{\mathrm{cm}}^{3}$ cubic PMMA phantom was placed in the ULF-MRI scanner, such that the phantom was centered at the treatment isocenter. A Semiflex detector (Type 31010, PTW, Freiburg, Germany), connected to a Unidos Webline (PTW, Freiburg, Germany) electrometer was positioned at the center of the PMMA phantom in a CNC machined opening. Charges collected were corrected by temperature and pressure as recommended by TRS398 rev 1 [25] and converted to dose to water using in-house established cross calibration factors for protons. The Semiflex detector was cross-calibrated in-house against a Farmer chamber (Type 30013, PTW, Freiburg, Germany), which had previously been calibrated in ${}^{60}\mathrm{Co}$ at the Austrian standards laboratory and corrected for proton beam quality. Following the TRS398 substitution method, both chambers were sequentially irradiated under identical conditions in a 10 × 10 cm^2^ monoenergetic proton field with an energy of 179.2 MeV at a water-equivalent depth of 2 cm. No correction due to the presence of a magnetic field was performed. The standard deviation of the mean (STOM) was calculated.

A monoenergetic 2D field, covering $6\times 6\;{\mathrm{cm}}^{2}$ with 2 mm spot spacing employing 148.2 MeV protons was generated. In addition, a 3D dose distribution, covering a $4\times 4\times 4\;{\mathrm{cm}}^{3}$ cubic volume, was optimized such that the center of the SOBP was located at a depth of 3 cm in the center of the PMMA phantom. Both plans delivered approximately 2 Gy to the ionization chamber.

Potential influences of ongoing MR imaging sequences on dosimetric measurements were evaluated. The monoenergetic 2D plan was irradiated 10 times during ongoing imaging sequences as well as 10 times without imaging. In addition, the 3D SOBP was irradiated 3 times with and without ongoing imaging. Due to the PMMA phantom, the sequences did not produce useful images and were therefore not evaluated.

## 3 Results

Electromagnetic interference measurements in the treatment room revealed considerable presence of EMI. Grounding and subsequent shielding restored noise levels to near baseline levels (Table 1) and considerably improved subjective visual image quality (Fig. 2). Minor EMI is noticeable for the Grounding and Shield configuration as highlighted by the red arrows. The addition of the inner shield improved the SNR by 20% compared to the configuration where the phantom was only grounded.

Image quality achieved in the irradiation room without beam delivery was comparable to laboratory conditions and no beam- or scanning magnet-induced artifacts were observed for either protons or carbon ions (Fig. 3). Beam delivery had no noticeable effect on geometric accuracy, which remained constant across all configurations. S–I measured 148 mm and L–R 150 mm, versus the physical phantom dimensions of 148 mm in both S–I and L–R. Neither the SNR across configurations nor the PSNR between reference and beam-delivery images changed noticeably.

**Fig. 2:**
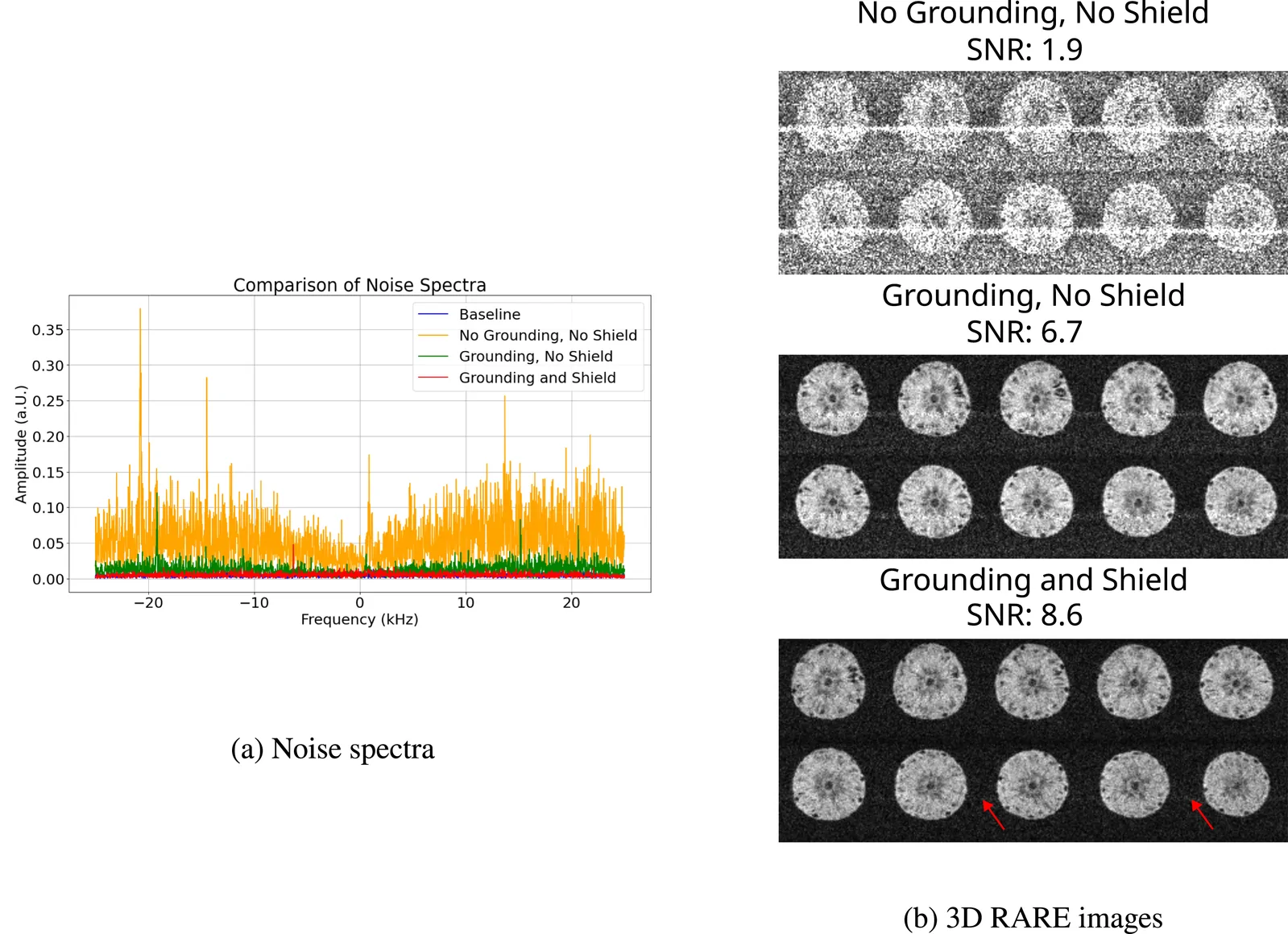
(a) Noise spectrum measurements for different EMI mitigation configurations. (b) 3D RARE images of the pineapple phantom under different EMI mitigation configurations. Minor EMI is noticeable for the Grounding and Shield configuration as highlighted by the red arrows.

**Table 1:** Measured noise level and Noise Factor for different EMI mitigation configurations. The Noise Factor is scaled to a 50 Ω baseline which was 188.5μV.

EMI mitigation	Noise level (μV)	Noise Factor
No Grounding, No Shield	2254.83	11.96
Grounding, No Shield	520.62	2.762
Grounding and Shield	204.57	1.09

Proton beam deflections at the measurement position 50 cm behind the isocenter ranged from 13.6 to 6.3 mm, depending on the energy, with only lateral offsets being present (Table 2). Spot displacements were similar for all spots and found to be independent of the individual spot position, indicating no additional influence of either the static magnetic field or the imaging sequence. The numerical solution of the beam path yielded comparable results and enabled calculation of the spot offset at isocenter due to the magnetic field, ranging from 1.7 mm at 62 MeV to 0.9 mm at 252 MeV (Table 2).

**Fig. 3:**
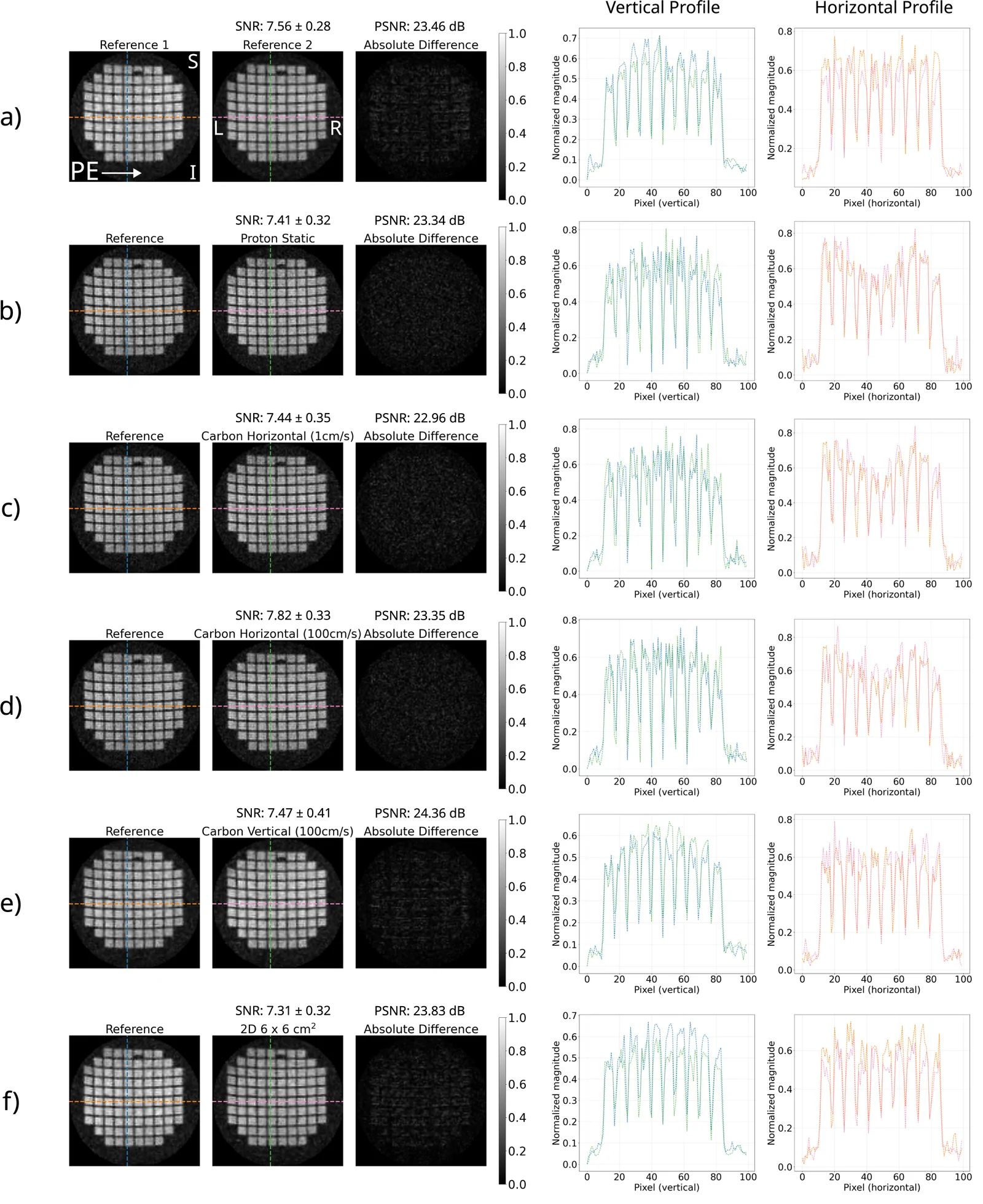
Comparison and difference images as well as SNR calculated over ten slices with standard deviation and normalized signal intensity for a single line in vertical and horizontal direction of the geometric phantom from top to bottom, (a) without irradiation, (b) with a central straight proton beam, (c) a carbon ion beam scanned horizontally with about 1 cm/s, (d) with a carbon ion beam scanned horizontally with about 100 cm/s, (e) with a carbon ion beam scanned vertically with about 100 cm/s, (f) with a carbon ion beam scanned over a monoenergetic 2D 6 × 6 cm^2^ with about 100 cm/s. The location of the vertical and horizontal intensity profile is marked in the image with according colors. The phase encoding (PE) direction and orientations (S-I and L-R) are indicated in (a) . A dark spot is visible where the 3D-printed phantom has residual air enclosed.

Similar lateral offsets were found for the 2D fields. Investigated parameters such as field size at 50% and 90% dose level, penumbra as well as symmetry agreed within 0.9%. No difference was observed for 2D homogeneity, as can also be seen on a dose difference map (Fig. 4(b)). No additional deformations were detected.

**Table 2:** Spot deflections of the individual spots of the 9 spot maps used, measured 50 cm downstream of the isocenter using the IBA Lynx detector (IBA Dosimetry, Schwarzenbruck, Germany), as well as calculated using the numerical solver at isocenter and the measurement position.

Proton energy	Position	Measured				Calculated
		Avg. lateral offset	StdDev	Avg. vertical offset	StdDev	lateral offset
[MeV]	[cm]	[mm]	[mm]	[mm]	[mm]	[mm]
62.4	50	13.6	0.07	0.1	0.2	13.7
148.2	50	8.5	0.05	0.1	0.1	8.1
252.7	50	6.3	0.08	0.0	0.1	6.0
62.4	0	–	–	–	–	2.1
148.2	0	–	–	–	–	1.3
252.7	0	–	–	–	–	0.9

For the 2D mono-energetic fields, proton doses measured with and without MR imaging agreed within 0.05%. The STOM was found to be 0.033% and 0.3% with and without imaging, respectively. A comparison to measurements performed without the ULF-MR scanner showed similar STOM, indicating no additional influence of the static magnetic field nor the imaging sequence.

**Fig. 4:**
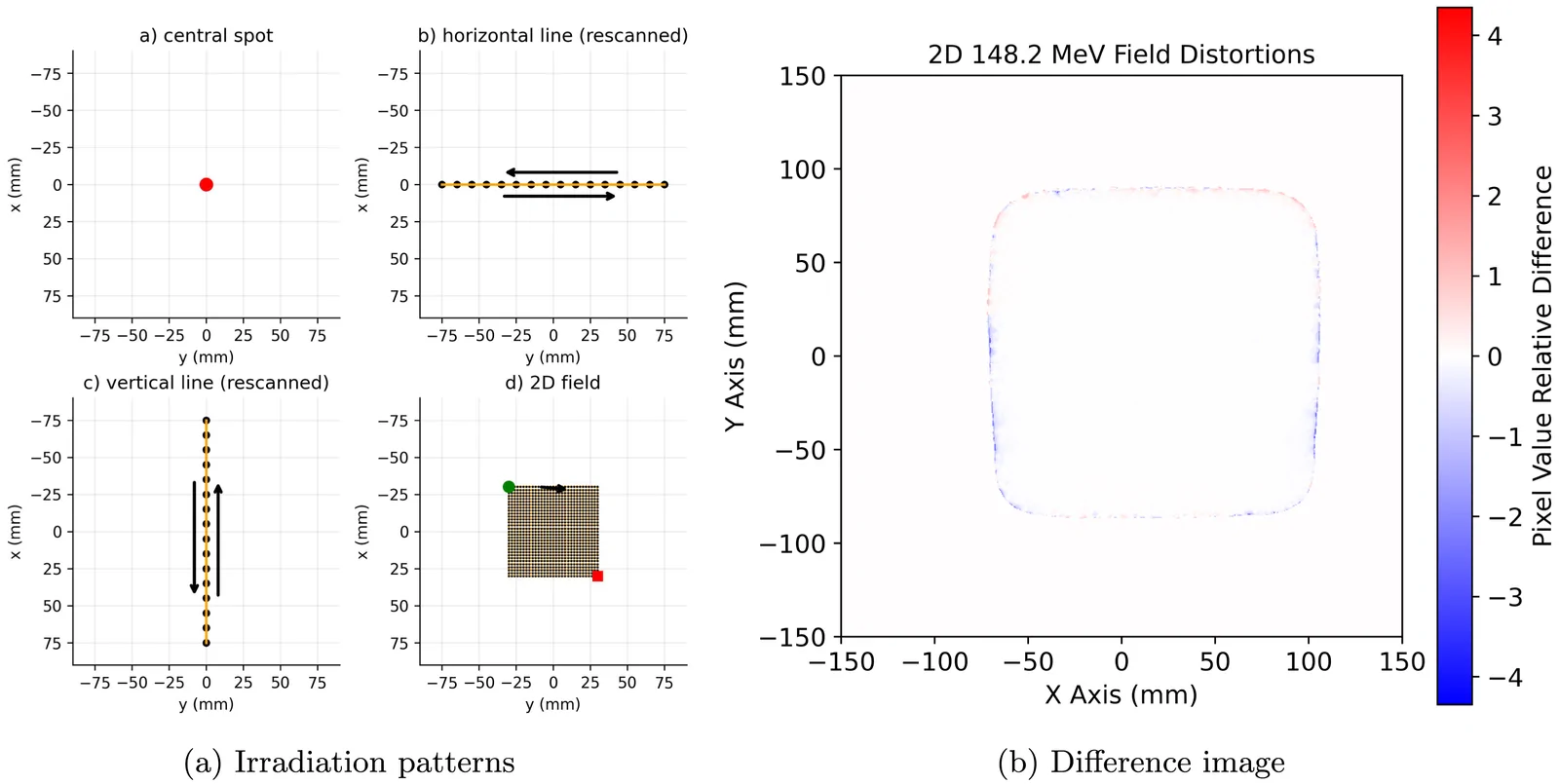
(a) Irradiation patterns used for imaging artifact investigation, (a) central spot with 252.7 MeV protons, (b) horizontal line using slow and fast scanning with 402.8 MeV/u carbon ions, (c) vertical line using slow and fast scanning with 402.8 MeV/u carbon ions, and (d) a 2D field of $6\times 6\;{\mathrm{cm}}^{2}$ with 2 mm spot spacing employing 148.2 MeV protons; (b) Difference images of $14\times 14\;{\mathrm{cm}}^{2}$ irradiation field using 148.2 MeV protons with and without the ULF-MR system, measured using the Lynx detector (IBA Dosimetry, Schwarzenbruck, Germany).

For the SOBP, the same behavior was found with doses agreeing within 0.02% and STOM to be 0.01% and 0.02% with and without imaging, respectively.

## 4 Discussion

This feasibility study demonstrates that a stable subjective visual image quality can be achieved with a ULF-MRI system in an ion-beam therapy environment without a dedicated shielding room when appropriate EMI mitigation strategies are applied. Despite substantial environmental electromagnetic interference, grounding and local shielding reduced noise levels close to baseline conditions, enabling reliable image acquisition.

Different particle types were employed to investigate interference mechanisms on MR imaging: (i) effects of the treatment beam itself and (ii) indirect effects related, for example, to beam scanning.

No such effects were observed, consistent with prior reports for high-field systems [26], [27] and confirmed here for ultra-low-field MRI.

Scanning-related effects are expected to be more pronounced for carbon ions due to their higher magnetic rigidity, requiring stronger magnetic field strength. To probe dynamic influences, two scanning speeds with alternating directions were applied, with the higher speed representing a worst-case scenario with the slower scanning speed closer to clinical routine.

The scanning magnets are located 7 m upstream of the isocenter and shielded by 2 m of concrete, substantially reducing stray fields at the imaging site. While such disturbances have been reported for a 0.25 T system under comparable conditions [4], the present results indicate an increased robustness of ultra-low-field MRI. However, sequence-dependent effects remain to be investigated. A proton therapy center with the scanning magnets located directly at the treatment nozzle reported higher effects for 0.25 T scanners [28] and should be evaluated for ultra-low-field MRI in future studies.

ULF-MRI systems are sensitive to $B_{0}$ field drifts due to temperature variations, which likely explain the deviations between reference images during the experimental campaign. In Fig. 3, the configurations with two reference measurements (a), Carbon Vertical (b), and 2D 6 × 6 cm^2^ (c) show the largest deviations. The difference in signal magnitude, as observed in the signal intensity plots, indicates a temperature drift causing off-resonance excitation and detection of the MRI signal, which would result in the signal intensity drop visible in the plots. Previous work characterizing MRI image artifacts during proton pencil beam scanning revealed that fringe field effects from the beam line caused ghosting artifacts in the phase-encoding direction for Gradient Echo (GRE) sequences [28]. No ghosting artifacts are noticeable in Fig. 3, which would be apparent in areas outside the phantom in the difference images. PSNR as well as SNR stays comparable across different beam configurations.

As expected, even the relatively small magnetic field strength of the ULF-MRI system induced measurable beam deflections, as the field was present over a relatively large area. At treatment isocenter, spot offsets were considerably smaller compared to higher field prototypes [3], which might allow the use of less advanced compensation methods. Other beam distortions as observed for a 0.25 T prototype [3] were not observed. A limitation of this study is that changes in Bragg-peak position were not measured because only negligible effects were expected at the low magnetic field strength and magnetic field volume investigated. Nevertheless, such changes may become more relevant in larger systems and should therefore be evaluated in future studies.

Dosimetric measurements exhibit no measurable impact of either static or dynamic magnetic and RF fields on dose determination in this setup. While the experimental configuration was not suitable for small magnetic-field-induced detector response changes, both these results and prior work [8] indicate such effects are negligible at this field strength.

This proof-of-principle study used a prototype ULF-MR system, imposing several limitations that are addressable with more advanced implementations. Imaging was limited to phantoms, and image quality was constrained by the absence of advanced reconstruction and correction methods. Acquisition parameters were not optimized and can be further improved for either speed or resolution. Hardware development is ongoing to approach the intrinsic SNR limit, which in this experiment was limited by a non-ideal low noise amplifier (LNA) that introduced noticeable broadband noise. A revised LNA design improved absolute SNR by a factor of 1.5 to 2 and will be evaluated in future experiments.

The chosen voxel size ($2\times 2\times 5\;{\mathrm{mm}}^{3}$) is close to the isotropic resolutions (∼2–3 mm^3^) currently employed for dose calculation in the treatment planning process. This suggests that the resulting images may retain sufficient spatial information for downstream applications such as dose recalculation. However, the suitability for such applications remains to be investigated in future studies.

The employed voxel size was inspired by current imaging protocols, where the in-plane resolution is higher than in the craniocaudal direction.

While the setup corresponds to a specific treatment room geometry, and results may vary across facilities, no fundamental limitations were identified. Overall, the findings indicate that ULF-MR systems can be further investigated for potential application in online MR-guided ion-beam therapy, with additional improvements (e.g. SNR, resolution, or visual image quality) expected from ongoing hardware and software development.

For the first time, an ultra-low-field MR scanner was successfully operated in an ion therapy research room without large external shielding structures and without compromising image quality or dose delivery, indicating its potential as an alternative approach for MR-guided proton therapy.

## CRediT authorship contribution statement

**Julia Pfitzer:** Writing – review & editing, Writing – original draft, Visualization, Methodology, Investigation, Formal analysis, Data curation, Conceptualization. **Hermann Scharfetter:** Writing – review & editing, Supervision, Resources, Methodology, Investigation, Funding acquisition, Conceptualization. **Panagiotis Antypas:** Writing – review & editing, Methodology, Investigation, Formal analysis, Data curation. **Dietmar Georg:** Writing – review & editing, Supervision, Resources. **Hermann Fuchs:** Writing – review & editing, Writing – original draft, Validation, Supervision, Resources, Project administration, Methodology, Investigation, Funding acquisition, Formal analysis, Data curation, Conceptualization.

## Declaration of competing interest

The authors declare that they have no known competing financial interests or personal relationships that could have appeared to influence the work reported in this paper.

## Appendix A Supplementary data

MMC S1 Feasibility of ultra-low-field MRI in an ion beam treatment room for online MR-guided particle therapy. MMC S1
