# Supplementary material for: Feasibility of ultra-low-field MRI in an ion beam treatment room for online MR-guided particle therapy
Source: Phys Imaging Radiat Oncol. 2026 Sep 26;41:101092. doi: 10.1016/j.phro.2026.101092 (PMC13659474; doi:10.1016/j.phro.2026.101092)
Supplement: Supplementary file 1 [file mmc1.pdf]

Supplementary Material

Feasibility of Ultra-Low-Field MRI for Online MR-Guided Particle Therapy: First Experimental Operation in an Ion Beam Treatment Room

Table S1: MRI Acquisition Parameters for EMI measurements

**Parameter**	**Value**
Sequence	3D RARE
Flip Angle	90°, 180°
TR/echo spacing/echo train length	500 ms / 20 ms / 5
Field-of-view	$160 \times 160 \times 120$ mm^3
Data points	$80 \times 80 \times 24$ (RO, PH1, PH2)
Voxel size	$2 \times 2 \times 5$ mm^3
Acquisition bandwidth	10 kHz
RF pulse length	250 μs / 500 μs
Trajectory	Cartesian Inside-out
Averages	1
Acquisition Time	approx. 3 minutes 20 seconds

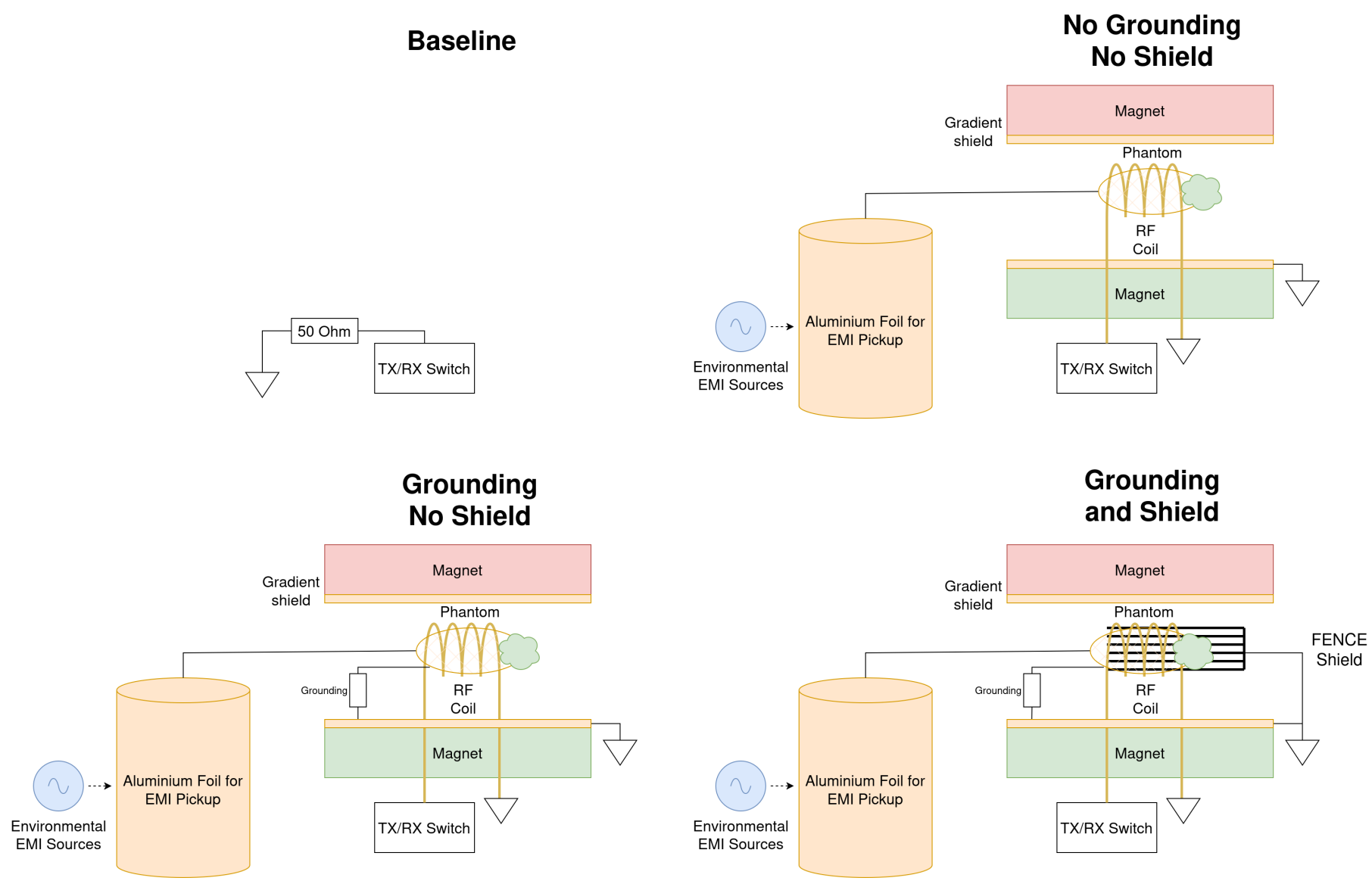

Figure S1: Setup for the EMI measurements. For the *Baseline* measurement the TX/RX switch was terminated with 50 Ω. In the *No Grounding, No Shield* configuration the pineapple for imaging was connected to a large aluminum foil surface placed outside the scanner for EMI pickup. The aluminum foil and pineapple were connected with a short cable and a copper needle was inserted into to pineapple. For the *Grounding, No Shield* configuration the pinapple was connected to RF ground using a copper needle and a 100 Ω series resistance to mimic a electrode-body interface. In the *Grounding and Shield* configuration an internal RF shield (Flexibel Electric Noise reduCtion Endo-shield / FENCE)[1] was added to the RF coil. PCB shields carried parallel copper strips with 1 mm width and 1 mm spacing. Solder connectors at the edges allowed multiple PCBs to be joined together for modular RF shield construction. The single-layer PCBs were fabricated with 18µm copper thickness on 25µm dielectric substrate (Polyimide) with a total thickness of 0.07mm.

Table S2: MRI Acquisition Parameters for phantom measurements. The phantom was filled with copper sulfate (1.5g/l) doped deionized water as MRI measurand.

**Parameter**	**Value**
Sequence	3D RARE
Flip Angle	90°, 180°
TR/echo spacing/echo train length	400 ms / 20 ms / 5
Field-of-view	$200 \times 200 \times 120$ mm^3
Data points	$100 \times 100 \times 24$ (RO, PH1, PH2)
Voxel size	$2 \times 2 \times 5$ mm^3
Acquisition bandwidth	20 kHz
RF pulse length	250 μs / 500 μs
Trajectory	Cartesian Inside-out
Averages	1
Acquisition Time	approx. 3 minutes 20 seconds

References

[1] Pfitzer J, Uecker M, Scharfetter H. FENCE: Flexible Electric Noise Reduction Endo-Shield for the Suppression of Electromagnetic Interference in Low-Field MRI. NMR Biomed. 2026 Apr;39(6). doi:10.1002/nbm.70287.
